# Representation of Women and Pregnant Women in HIV Research: A Limited Systematic Review

**DOI:** 10.1371/journal.pone.0073398

**Published:** 2013-08-23

**Authors:** Daniel Westreich, Molly Rosenberg, Sheree Schwartz, Geeta Swamy

**Affiliations:** 1 Department of Epidemiology, University of North Carolina at Chapel Hill, Chapel Hill, North Carolina, United States of America; 2 Department of Obstetrics and Gynecology, Duke University, Durham, North Carolina, United States of America; 3 Duke Global Health Institute, Duke University, Durham, North Carolina, United States of America; University of Toronto, Canada

## Abstract

**Background:**

HIV-related outcomes may be affected by biological sex and by pregnancy. Including women in general and pregnant women in particular in HIV-related research is important for generalizability of findings.

**Objective:**

To characterize representation of pregnant and non-pregnant women in HIV-related research conducted in general populations.

**Data Sources:**

All HIV-related articles published in fifteen journals from January to March of 2011. We selected the top five journals by 2010 impact factor, in internal medicine, infectious diseases, and HIV/AIDS.

**Study Eligibility Criteria:**

HIV-related studies reporting original research on questions applicable to both men and women of reproductive age were considered; studies were excluded if they did not include individual-level patient data.

**Study appraisal and synthesis methods.:**

Articles were doubly reviewed and abstracted; discrepancies were resolved through consensus. We recorded proportion of female study participants, whether pregnant women were included or excluded, and other key factors.

**Results:**

In total, 2014 articles were published during this period. After screening, 259 articles were included as original HIV-related research reporting individual-level data; of these, 226 were determined to be articles relevant to both men and women of reproductive age. In these articles, women were adequately represented within geographic region. The vast majority of published articles, 183/226 (81%), did not mention pregnancy (or related issues); still fewer included pregnant women (n=33), reported numbers of pregnant women (n=19), or analyzed using pregnancy status (n=9).

**Limitations:**

Data were missing for some key variables, including pregnancy. The time period over which published works were evaluated was relatively short.

**Conclusions and implications of key findings.:**

The under-reporting and inattention to pregnancy in the HIV literature may reduce policy-makers’ ability to set evidence-based policy around HIV/AIDS care for pregnant women and women of child-bearing age.

## Introduction

Worldwide, about half of those living with HIV and AIDS are women and the majority of those women are of child-bearing age. In sub-Saharan Africa, where the HIV disease burden is most severe, more than 60% of adults living with HIV are women [1], and risk of HIV acquisition is concentrated among women of reproductive age. In South Africa, in particular, young women have more than three times the estimated prevalence of HIV infection compared with young men [2,3]. Prevalence of HIV is even higher among young women in antenatal care [3], and pregnancy is common among both HIV-infected women [4–7] and women at risk of HIV infection [4,8] throughout sub-Saharan Africa.

A substantial amount of HIV research to date has concentrated on prevention of mother to child transmission (PMTCT), as well as on the impact of PMTCT on subsequent maternal responses to highly active antiretroviral therapy (HAART) [9–12]. However, such research typically concentrates on pregnant women exclusively with primary outcomes that are not focused on maternal health but rather infant or child outcomes. Far less research is performed treating pregnancy either as an exposure (comparing pregnant to non-pregnant women) or as a confounder, modifier, or mediator of main effects [13].

Including pregnant women in non-pregnancy specific studies and assessing pregnancy as an exposure is important as pregnancy may influence HIV-related outcomes through hormonal, immunological or other physiological changes to the female body, or by altering drug pharmacokinetics [14,15]. Research around the impact of pregnancy on HIV progression and survival has produced varied findings [16–18]. Other studies have suggested that women are more susceptible to HIV-infection during pregnancy, as well as more likely to transmit HIV to partners [19,20]. Although evidence in these areas is limited and inconsistent [21], these examples reinforce the idea that pregnancy may impact HIV-related outcomes and should be considered when conducting HIV-related research.

To be relevant, HIV/AIDS research as a whole should be performed in populations with proportional composition to those affected by the disease. While all NIH-funded clinical trials were required to include women starting in 1993 with the NIH Revitalization Act (PL 103-43) [22], concerns remain about the representation of women in trials generally [23–26]. No such requirements apply to pregnant women, who have been routinely excluded from medical research in general and clinical trials in particular [27]. No requirements exist for the inclusion of either women or pregnant women in observational research, nor do related guidelines such as STROBE (Strengthening the Reporting of Observational Studies in Epidemiology) mention this issue [28].

It is at present unknown if women are adequately represented in HIV/AIDS research, in either observational or experimental studies. Informally, we have observed that HIV/AIDS research within general populations typically fails to meaningfully address pregnancy, either by excluding pregnant women where no such exclusion is justifiable, or by failing to address pregnancy in a population which includes numerous women of reproductive age. Such exclusions, if widespread, may lead to biased research findings. Here, we sought to formalize these perceptions by assessing the frequency with which pregnant women, and women in general, are included or excluded from representative publications in the peer-reviewed HIV/AIDS literature dealing with research conducted in general populations – that is, in studies dealing with questions relevant to both men and women generally.

## Materials and Methods

### Types of studies and search strategy

To quantify how well women and pregnant women were represented among representative current studies of HIV, we examined all HIV-related articles officially published between January and March 2011 from fifteen high impact journals which publish HIV/AIDS research. Using the Journal Citation Report (Science) [29], we identified the top five journals publishing primarily original research by 2010 impact factor within each of three categories: general and internal medicine; infectious diseases; and HIV/AIDS. The first group of journals comprised the top five journals listed by impact factor in the journal category “Medicine, General & Internal”; the latter two groups of journals were both drawn from the category “Infectious Diseases”. The AIDS-specific journals were the top five journals by impact factor in this category with “HIV” or “AIDS” in the journal title; the infectious diseases journals were the top five journals by impact factor in the category after removal of the AIDS-specific journals. As our goal was to assess the mainstream AIDS literature and research relevant to the general population, we deliberately did not consider journals focused on obstetrics and gynecology or women’s health.

We conducted the search within these fifteen journals over a publication period of three months using PubMed.gov and the journal websites. We restricted the search to remove common publication types which do not include original research: historical articles, news, editorials, non-research-letters, comments, reviews, meta-analyses, case reports, and patient education handouts (accounting for slight variations in naming conventions among journals). We restricted further to the studies related to HIV using the following search terms: “HIV”, “AIDS”, “retrovir*”, and “human immunodeficiency virus” (where * indicates a match to any text string, so “retrovir*” will match, among other things, “antiretroviral”).

All articles identified by the above search criteria were considered. Articles were independently and doubly reviewed and abstracted; discrepancies in abstracted data were resolved through consensus. Articles were removed from further analysis if they were unrelated to HIV (e.g., involving an alternative retrovirus); not original research (e.g., commentaries or invited reviews); or ecological, modeling, surveillance, or other studies which did not report or analyze individual-level data. If articles referred to a previously defined study population (e.g., an observational study performed within a randomized trial population), the original referenced description of the population was identified and used to abstract additional information if necessary. The remaining studies were then screened for those with research questions relevant to pregnant women only, primarily children under 11, or men only. Studies which addressed a research question which was not male-specific, but which was done in an all-male population, were not considered as research questions relevant to men only.

### Data collection and analysis

Among studies with research questions potentially relevant to men and women of reproductive age, we abstracted data on the participation and analysis of women and pregnant women in the study and article. Specifically, we recorded: the proportion of study participants who were women; whether pregnant women were included or excluded, or if no data were collected on pregnancy status; and whether or not the article mentioned pregnancy at all (using search strings: “preg”, “matern”, “grav”, “natal”, and “child”). For articles which did not report proportion women or inclusion/exclusion criteria involving pregnancy, we contacted authors by email to ascertain this information. Additional characteristics abstracted from the articles included: observational versus experimental study type, whether or not the study used a previously defined study population, country and region in which the study was performed, sample size, and age range of participants.

To contextualize the inclusion of women in research, we searched UNAIDS, CDC, and US Government websites to obtain estimates of the proportion female among persons living with HIV and AIDS in each major world region. We compared these estimates to the proportion of female study subjects included in the reviewed manuscripts by region (calculated as total female participants within all studies in the same region divided by total participants from all studies in that region). To explore the possibility that representation of women and pregnant women may differ by type of research, we examined the participation of women and pregnant women stratified by whether the study was experimental or observational.

Two main analyses were conducted using simple descriptive statistics: first, the representation of women in studies. Second, the inclusion or exclusion of pregnant women in studies.

## Results

### Studies selected

**Figure 1:**
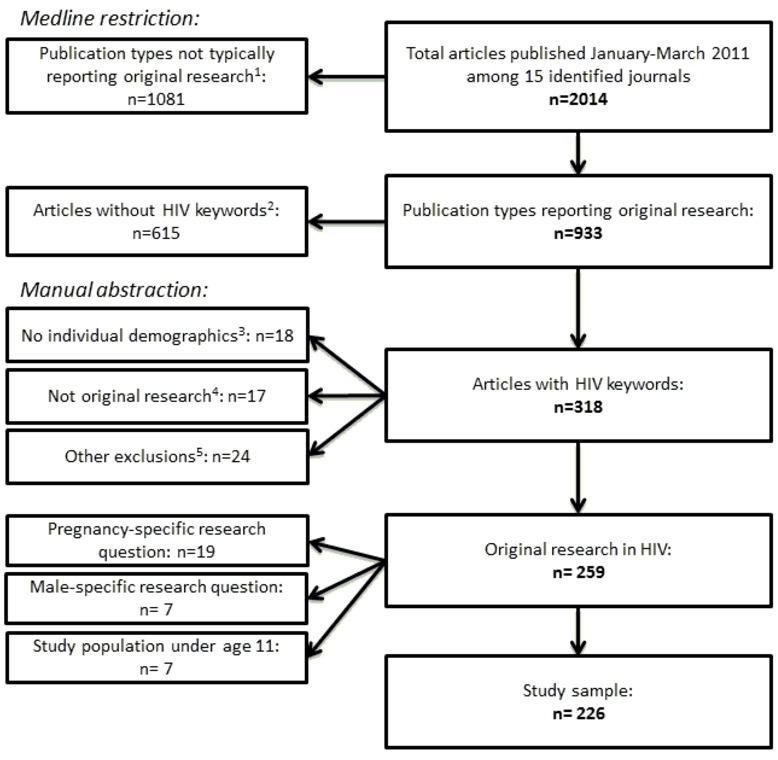
Flowchart of included studies. ^1^Historical articles, news, editorials, letters, comments, case reports, reviews, patient education handouts, meta-analyses. ^2^Keywords: HIV, AIDS, anti-retrovir*, antiretrovir*, retrovir*, human immunodeficiency virus. ^3^Ecological studies, surveillance studies, animal studies, modeling studies. ^4^Commentary, reviews, invited articles. ^5^Other retroviruses, studies of non-HIV related outcomes in HIV-negative populations, study of clinicians/service providers, studies in non-human subjects, blood donation screening study.

Table 1 lists the fifteen journals identified for review. Of 2,014 total articles published in the identified journals from January to March 2011, a total of 318 articles contained HIV-related keywords. Of these 318 studies, 59 were removed because they contained no individual-level demographic data (n=18), they were not original research (n=17), or for other reasons (n=24), listed in Figure 1. Of the 259 remaining HIV-related articles, 19 addressed a pregnancy-specific research question; 7 addressed a male-specific research question; and 7 included a study population of all (or large majority) children under age 11. We judged that the remaining 226 articles *could have* included men and both pregnant and non-pregnant women. These 226 studies comprise our data set for subsequent analyses and are listed in appendix File S1. These studies were assessed by region: 152 (67%) studies were from North America, Western or Central Europe, while 32 (14%) were from sub-Saharan Africa.

**Table 1:** Journals surveyed and original HIV literature included in each journal, January-March 2011.

**Subject**	**Title**	**Total Articles**	**Research Articles**	**HIV/AIDS Articles**
*General Medicine*	The New England Journal of Medicine	340	112	1
	The Lancet	373	88	7
	JAMA: The Journal of the American Medical Association	235	89	4
	Annals of Internal Medicine	148	42	0
	PLoS Medicine	22	17	5
*Infectious Disease*	The Lancet Infectious Diseases	51	13	3
	Clinical Infectious Diseases	193	105	29
	Emerging Infectious Diseases	137	73	3
	Journal of Infectious Diseases	125	100	30
	Clinical Microbial Infections	82	61	3
*HIV/AIDS*	AIDS	127	93	93
	JAIDS: Journal of Acquired Immune Deficiency Syndromes	79	60	60
	HIV Medicine	24	21	21
	AIDS Patient Care	32	21	21
	AIDS Research Human Retroviruses	46	39	38
		**2014**	**933**	**318**

### Representation of women

Of the 226 evaluable studies, 196 had complete sample size and percentage female available for analysis. In these 196 studies, women were generally represented at or above the estimated within-region normative values (Table 2). Exceptions were the Caribbean, where we evaluated only a single study involving 7 people, and Asia/Pacific, where representation of women was slightly below normative levels. We note that these by-region normative values may in fact understate the expected percentage female among women of reproductive age, because in many settings (including sub-Saharan Africa) young women are at higher risk of HIV infection than young men.

**Table 2:** Number of subjects and proportion female subjects among 226 analyzed articles, by region.

**Regional information:**					**Among studies analyzed:**	
**Region**	**% female among PLWHA^‡^**	**% pregnant among PLWHA^†^**	**# studies**	**# analyzed**	**# subjects**	**% subjects female**
N. Am/Europe	25%	<0.4%	152	131	252,518	33.1%
Sub-Saharan Africa	60%	5.8%	32	32	42,879	66.7%
Latin Am	36%	1.3%	11	9	15,996	71.4%
Asia/Pacific	35%	1.5%	10	8	21,817	33.6%
Caribbean	53%	3.2%	1	1	7	42.9%
Eastern Europe	30-40%	1.3%	1	0	n/a	n/a
Multi-region	n/a	n/a	19	15	12,869	19.1%
Total	50%	4.4%	226	196	346,086	38.5%

PLWHA: people living with HIV and AIDS

^‡^ Normative values from [56–58]

^†^ Estimated from [59,60]

Despite the fact that women were represented adequately within region, they were underrepresented overall: while 50% of people living with HIV and AIDS worldwide are women, only 38.5% of those in these studies were woman. This is due primarily to the large proportion of studies and study subjects (73%) from North America and Europe, where women are underrepresented compared to the total world population of people living with HIV and AIDS (although women were *overrepresented* in studies from this region, see Table 2). Of the 226 studies relevant to men and women, 11 (5%) included no women at all, while 9 (4%) included only women.

### Representation of pregnancy and pregnant women

Only 43 (19%) mentioned pregnancy at any point in the article, while 81% did not mention pregnancy (Table 3). A total of 33 studies (15%) included pregnant women in the study population, while 104 (46%) included no pregnant women. In 89 studies (39%), it was unknown whether pregnant women were included in their analysis, because no information was present in the manuscript and either authors stated by email that they did not know whether pregnant women were included in their analysis (34 studies), or authors did not respond to email (55 studies).

**Table 3:** Characteristics of 226 studies relevant to men and women of reproductive age, overall and by study design.

**Characteristics**			**Total n=226**	**By study design:**		**Among observational studies only:**	
				**Experimental n=25**	**Observational n=151**	**Previous cohort n=104**	**Original cohort n=47**
Included no women			11 (5%)	4 (16%)	6 (4%)	4 (4%)	2 (4%)
Pregnancy not mentioned			183 (81%)	17 (68%)	122 (81%)	84 (81%)	38 (81%)
Pregnancy inclusion							
	Inclusion		33 (15%)	2 (8%)	26 (17%)	22 (21%)	4 (9%)
	Non-inclusion		104 (46%)	20 (80%)	67 (44%)	45 (43%)	22 (47%)
		Exclusions^‡^	61 (27%)	16 (64%)	39 (26%)	31 (30%)	8 (17%)
	Unknown		89 (39%)	3 (12%)	58 (38%)	37 (36%)	21 (45%)

All figures are n (%); numbers do not sum to 100% because of rounding.

^‡^ Either explicit or implicit exclusions, see text.

Of the 104 studies which did not include pregnant women, 25 (including 7 trials) *explicitly excluded* pregnant (and/or recently pregnant, or lactating women). There were a variety of reasons (explicit and implicit) for these exclusions (although some studies gave no reasons, e.g. [30–32]). Some studies noted differences in: management of pregnant women [33]: study sites at which pregnant women are seen [34]; or the potential for pregnancy related physiologic changes relevant to study aims [35]. Numerous studies [36–40] examined questions related to efavirenz, a possible reason for the exclusion of pregnant women, though few if any of these studies were explicit about reasons for the exclusion. Six (related) observational studies of current users of buprenorphine [41–46] excluded pregnant women. Three studies which excluded pregnancy were H1N1 vaccine studies [47–49]; one identified pregnant women as being at particularly high risk from H1N1 before then excluding pregnant women [49].

Another 36 studies *implicitly excluded* pregnant women. Implicit exclusions took several forms. Among these were studies which performed secondary analysis of data from a trial which had, itself, excluded pregnant women; studies which mentioned pregnancy exclusions on the trial registration website, but not in the paper; studies which used an all-male cohort or analysis to answer a research question which was not male-specific; or studies for which exclusions were only communicated by email, not in the paper. Two studies [50,51] examined an outcome of incident pregnancy but excluded prevalent pregnancies; we did not count these two studies as excluding pregnant women.

Of the 33 studies which did include pregnant women as part of their study population, 16 did not mention this in their text; the information was obtained by author query. A total of 12 studies out of the 226 assessed (7%) reported the actual number of incident and/or prevalent pregnancies in the article text; another 7 studies reported the number of pregnancies via author correspondence. Only 9 studies used pregnancy status in analysis: as a covariate (n=3), an outcome (n=2), subgroup (n=2), exposure (n=1), or describing distribution of pregnancy status between arms of a trial (n=1).

### Experimental vs. observational studies

We then limited our 226 studies to 176 population-based experimental and observational studies, excluding laboratory, genetic, diagnostic testing studies, and ambiguous study designs; there were 25 experimental studies and 151 observational studies. In this subset, women continued to be represented adequately in both experimental and observational studies when considered by region.

Experimental studies were somewhat more likely to mention pregnancy (8/25 experimental vs. 29/151 observational, χ^2^ p=0.15), but less likely to include pregnant women (2/25 vs. 27/151, χ^2^ p=0.22); neither result was statistically significant. Experimental studies were more likely to exclude pregnancy (all exclusions: 16/25 experimental vs. 39/151 observational, χ^2^ p<0.001). In studies where pregnancy was mentioned, experimental studies were more likely to exclude pregnancy as well (7/8 vs. 15/29; χ^2^ p=0.068).

### Original observational cohort studies

Among the 151 observational studies noted above, only 47 were performed in original cohorts, rather than being nested in other studies or trials. Of these, 9 (19%) mentioned pregnancy; 4 (9%) included pregnant women; and 8 (17%) excluded pregnant women explicitly or implicitly.

## Discussion

Women comprise 50% of those living with HIV and AIDS throughout the world, and women of reproductive age are at highest risk of HIV infection among those women. In Sub-Saharan African, where the heterosexual epidemic is most heavily concentrated, pregnancy is common among HIV-positive women, especially after the initiation of HAART [4–7]. In this systematic review of a sample of high impact HIV-related scientific literature, we found that women appear to be adequately represented in HIV/AIDS research conducted in the general population. Of concern, however, we also found that pregnant women are underrepresented in studied populations and that the great majority of HIV literature does not consider the presence or impact of pregnancy in their populations.

While a relatively low percentage of people living with HIV and AIDS are pregnant at a given time (Table 2), over time a large percentage of HIV-positive women are likely to experience pregnancy [4,50,52]. Given the importance of pregnancy, we would argue that more papers should be at least commenting on pregnancy in their cohort. Not only did 81% of papers not even mention pregnancy (or related terms) in their papers, we found from email correspondence that many investigators had no idea how many – indeed, if any – pregnant women were included in their study. UNAIDS has said that researchers “should recruit women… including those who may become pregnant, be pregnant or be breastfeeding” into clinical trials, since those women should be the “recipients of future safe and effective biomedical HIV prevention interventions.” [27,53] This has not yet come to pass: tellingly, the CONSORT website’s example on how to describe eligibility criteria for a trial is of an HIV trial which excludes pregnant women [54,55]. An even stronger case can be made for the inclusion of pregnant women in observational studies, which – unlike many trials – rarely introduce interventions such as new pharmacological agents which may pose fetal risks. Indeed, the fact that many RCTs systematically exclude pregnant women, often for good reason [27], means that it is even more important for observational studies to collect data from this group in order to better understand the safety of drugs, interventions, and outcomes for women exposed to pregnancy. Thus, we hoped to see better inclusion of pregnant women in observational studies than in experimental studies; inclusion was indeed higher in observational studies but remained low overall at 17%.

Many observational studies are themselves nested within “parent” cohorts or populations, a set of studies of which 21% included pregnant women. This puts the question of pregnancy out of the hands of investigators, especially when the parent cohort is a trial population and thus may well exclude pregnancy by design. While the use of existing data is tempting, we would urge future investigators to consider more carefully whether those data have exclusion criteria which make them inappropriate for the study question at hand. We would also urge investigators to do a more complete job of explaining the specific exclusion criteria in the parent study, and to specify the impact of these criteria on the generalizability of study findings.

Among original (non-nested) observational studies, only 9% included pregnant women: thus, there is a clear need for closer attention to pregnancy-related issues among researchers building new cohorts. Some of these studies were open to both non-pregnant and pregnant women, but enrolled only males and/or non-pregnant women. Thus, care must be taken in the design and recruitment stage to ensure adequate participant recruitment at appropriate levels for important subgroups.

Although women were underrepresented in the reviewed manuscripts, comprising less than 40% of the total subjects in these studies, women were adequately represented within region, e.g., the proportion of women subjects in studies from sub-Saharan Africa exceeded the estimated underlying proportion of women living with HIV and AIDS in that region. These apparently contradictory findings are due to the overrepresentation of the US/Europe among studies (152/226, 67%) and subjects (73% of total participants). In contrast, only 14% of the studies and 12% of subjects were from sub-Saharan Africa, where approximately two-thirds of all HIV-positive individuals reside.

There were limitations of this work. The review was conducted over a relatively short time period (three months, January through March 2011) and a set of fifteen journals which, while high impact and relevant to the HIV field, may not perfectly represent all population-based HIV research. It is possible that publications in lower-impact journals are including pregnant women in higher numbers. However, pregnancy is a significant and common issue among HIV-infected women and therefore a “high-impact” concern; the absence of pregnancy from high-impact publications is therefore problematic regardless of representation elsewhere. Laboratory-based studies were often hard to abstract data from because they frequently lack demographic data: however, we would argue that it would enhance reports of laboratory work to report basic demographics, including age, gender, and pregnancy status of the samples along with laboratory findings. Finally, there were substantial missing data for some variables, e.g., 39% of the 226 studies had no information available regarding pregnancy (Table 3). However, it seems clear that if pregnant women were not enumerated in results or mentioned in an article at all, that pregnancy was not being considered in the analysis, and that pregnancy data are not reaching the audience who make decisions about HIV/AIDS care for pregnant women and women of child-bearing age.

There are numerous challenges to including pregnancy in observational and well as experimental research, among them the time-varying nature of pregnancy, potential costs of laboratory testing, potential misreporting of pregnancy, and additional human subjects protections for the inclusion of pregnant women in research. Nonetheless, evidence-based public health policy necessitates research within those populations to which interventions will be targeted. In their guidance document, “Ethical considerations in biomedical HIV prevention trials”, UNAIDS/WHO states:

Researchers and trial sponsors should recruit women into clinical trials in order to verify safety and efficacy from their standpoint… since women throughout the life span, including those who may become pregnant, be pregnant or be breastfeeding, should be recipients of future safe and effective biomedical HIV prevention interventions [53].

It is clear that this statement applies to observational as well as experimental research efforts. In particular, observational studies can and should specifically assess the impact of interventions in populations that have been previously excluded from trial research, in order to better understand the generalizability of findings. Current HIV research in general populations, both experimental and observational, appears to adequately represent women in general, but appears to be insufficiently including, reporting, and analyzing pregnant women. This is both an ethical, and a scientific, issue.

## Supporting Information

File S1 **Bibliography of 226 studies considered in Table 2.**
(DOCX)
