# Supplementary material for: Representation of Women and Pregnant Women in HIV Research: A Limited Systematic Review
Source: PLoS One. 2013 Aug 23;8(8):e73398. doi: 10.1371/journal.pone.0073398 (PMC3751870; doi:10.1371/journal.pone.0073398)
Supplement: File S1 — Bibliography of 226 studies considered in Table 2. (DOCX) [file pone.0073398.s001.docx]

**S1. Bibliography of 226 studies considered in Table 2.**

1. Abbate I, Vlassi C, Rozera G, Bruselles A, Bartolini B, et al. (2011) Detection of quasispecies variants predicted to use CXCR4 by ultra-deep pyrosequencing during early HIV infection. AIDS 25: 611-617.

2. Achenbach CJ, Cole SR, Kitahata MM, Casper C, Willig JH, et al. (2011) Mortality after cancer diagnosis in HIV-infected individuals treated with antiretroviral therapy. AIDS 25: 691-700.

3. Addo MM, Altfeld M, Brainard DM, Rathod A, Piechocka-Trocha A, et al. (2011) Lack of detectable HIV-1-specific CD8(+) T cell responses in Zambian HIV-1-exposed seronegative partners of HIV-1-positive individuals. J Infect Dis 203: 258-262.

4. Agwu AL, Jang SS, Korthuis PT, Araneta MR, Gebo KA (2011) Pregnancy incidence and outcomes in vertically and behaviorally HIV-infected youth. JAMA 305: 468-470.

5. Altice FL, Bruce RD, Lucas GM, Lum PJ, Korthuis PT, et al. (2011) HIV treatment outcomes among HIV-infected, opioid-dependent patients receiving buprenorphine/naloxone treatment within HIV clinical care settings: results from a multisite study. J Acquir Immune Defic Syndr 56 Suppl 1: S22-32.

6. Amiel C, Charpentier C, Desire N, Bonnard P, Lebrette MG, et al. (2011) Long-term follow-up of 11 protease inhibitor (PI)-naive and PI-treated HIV-infected patients harbouring virus with insertions in the HIV-1 protease gene. HIV Med 12: 138-144.

7. Angel JB, Routy JP, Tremblay C, Ayers D, Woods R, et al. (2011) A randomized controlled trial of HIV therapeutic vaccination using ALVAC with or without Remune. AIDS 25: 731-739.

8. Arruda E, Simoes L, Sucupira C, Medeiros M, Arruda E, et al. (2011) Short communication: intermediate prevalence of HIV type 1 primary antiretroviral resistance in Ceara State, Northeast Brazil. AIDS Res Hum Retroviruses 27: 153-156.

9. Baeten JM, Lingappa J, Beck I, Frenkel LM, Pepper G, et al. (2011) Herpes simplex virus type 2 suppressive therapy with acyclovir or valacyclovir does not select for specific HIV-1 resistance in HIV-1/HSV-2 dually infected persons. J Infect Dis 203: 117-121.

10. Bakanda C, Birungi J, Mwesigwa R, Ford N, Cooper CL, et al. (2011) Association of aging and survival in a large HIV-infected cohort on antiretroviral therapy. AIDS 25: 701-705.

11. Baker JV, Neuhaus J, Duprez D, Kuller LH, Tracy R, et al. (2011) Changes in inflammatory and coagulation biomarkers: a randomized comparison of immediate versus deferred antiretroviral therapy in patients with HIV infection. J Acquir Immune Defic Syndr 56: 36-43.

12. Banasch M, Frank J, Serova K, Knyhala K, Kollar S, et al. (2011) Impact of antiretroviral treatment on (13) C-methionine metabolism as a marker of hepatic mitochondrial function: a longitudinal study. HIV Med 12: 40-45.

13. Banerjee S, McCutchan JA, Ances BM, Deutsch R, Riggs PK, et al. (2011) Hypertriglyceridemia in combination antiretroviral-treated HIV-positive individuals: potential impact on HIV sensory polyneuropathy. AIDS 25: F1-6.

14. Bansi L, Smith C, Phillips A, Kirk S, Geretti AM, et al. (2011) The impact of HIV drug resistance testing on changes to treatment. AIDS 25: 603-610.

15. Bassett IV, Chetty S, Giddy J, Reddy S, Bishop K, et al. (2011) Screening for acute HIV infection in South Africa: finding acute and chronic disease. HIV Med 12: 46-53.

16. Baum MK, Sales S, Jayaweera DT, Lai S, Bradwin G, et al. (2011) Coinfection with hepatitis C virus, oxidative stress and antioxidant status in HIV-positive drug users in Miami. HIV Med 12: 78-86.

17. Beachler DC, Gellert LL, Jacobson LP, Ambinder RF, Breen EC, et al. (2011) Kaposi sarcoma-associated herpesvirus serum DNA and antibodies not associated with subsequent non-Hodgkin lymphoma risk. J Acquir Immune Defic Syndr 56: 188-192.

18. Bickel M, von Hentig N, Wieters I, Khaykin P, Nisius G, et al. (2011) Immune response after two doses of the novel split virion, adjuvanted pandemic H1N1 influenza A vaccine in HIV-1-infected patients. Clin Infect Dis 52: 122-127.

19. Bizinoto MC, Leal E, Diaz RS, Janini LM (2011) Loci polymorphisms of the APOBEC3G gene in HIV type 1-infected Brazilians. AIDS Res Hum Retroviruses 27: 137-141.

20. Blackard JT, Welge JA, Taylor LE, Mayer KH, Klein RS, et al. (2011) HIV mono-infection is associated with FIB-4 - A noninvasive index of liver fibrosis - in women. Clin Infect Dis 52: 674-680.

21. Boue F, Reynes J, Rouzioux C, Emilie D, Souala F, et al. (2011) Alpha interferon administration during structured interruptions of combination antiretroviral therapy in patients with chronic HIV-1 infection: INTERVAC ANRS 105 trial. AIDS 25: 115-118.

22. Brigido LF, Ferreira JL, Almeida VC, Rocha SQ, Ragazzo TG, et al. (2011) Southern Brazil HIV type 1 C expansion into the state of Sao Paulo, Brazil. AIDS Res Hum Retroviruses 27: 339-344.

23. Briongos Figuero LS, Bachiller Luque P, Palacios Martin T, Gonzalez Sagrado M, Eiros Bouza JM (2011) Assessment of factors influencing health-related quality of life in HIV-infected patients. HIV Med 12: 22-30.

24. Brumme ZL, Li C, Miura T, Sela J, Rosato PC, et al. (2011) Reduced replication capacity of NL4-3 recombinant viruses encoding reverse transcriptase-integrase sequences from HIV-1 elite controllers. J Acquir Immune Defic Syndr 56: 100-108.

25. Bua A, Molicotti P, Ruggeri M, Madeddu G, Ferrandu G, et al. (2011) Interferon-gamma release assay in people infected with immunodeficiency virus. Clin Microbiol Infect 17: 402-404.

26. Bunupuradah T, Kosalaraksa P, Puthanakit T, Mengthaisong T, Wongsabut J, et al. (2011) Monoboosted lopinavir/ritonavir as simplified second-line maintenance therapy in virologically suppressed children. AIDS 25: 315-323.

27. Bygrave H, Ford N, van Cutsem G, Hilderbrand K, Jouquet G, et al. (2011) Implementing a tenofovir-based first-line regimen in rural Lesotho: clinical outcomes and toxicities after two years. J Acquir Immune Defic Syndr 56: e75-78.

28. Carrico AW, Riley ED, Johnson MO, Charlebois ED, Neilands TB, et al. (2011) Psychiatric risk factors for HIV disease progression: the role of inconsistent patterns of antiretroviral therapy utilization. J Acquir Immune Defic Syndr 56: 146-150.

29. Carsenti-Dellamonica H, Saidi H, Ticchioni M, Guillouet de Salvador F, Dufayard Cottalorda J, et al. (2011) The suppression of immune activation during enfuvirtide-based salvage therapy is associated with reduced CCR5 expression and decreased concentrations of circulating interleukin-12 and IP-10 during 48 weeks of longitudinal follow-up. HIV Med 12: 65-77.

30. Castelnuovo B, Kiragga A, Kamya MR, Manabe Y (2011) Stavudine toxicity in women is the main reason for treatment change in a 3-year prospective cohort of adult patients started on first-line antiretroviral treatment in Uganda. J Acquir Immune Defic Syndr 56: 59-63.

31. Catano G, Chykarenko ZA, Mangano A, Anaya JM, He W, et al. (2011) Concordance of CCR5 genotypes that influence cell-mediated immunity and HIV-1 disease progression rates. J Infect Dis 203: 263-272.

32. Chaiyachati K, Hirschhorn LR, Tanser F, Newell ML, Barnighausen T (2011) Validating five questions of antiretroviral nonadherence in a public-sector treatment program in rural South Africa. AIDS Patient Care STDS 25: 163-170.

33. Chan PA, Tashima K, Cartwright CP, Gillani FS, Mintz O, et al. (2011) Short communication: Transmitted drug resistance and molecular epidemiology in antiretroviral naive HIV type 1-infected patients in Rhode Island. AIDS Res Hum Retroviruses 27: 275-281.

34. Chang J, Lindsay RJ, Kulkarni S, Lifson JD, Carrington M, et al. (2011) Polymorphisms in interferon regulatory factor 7 reduce interferon-alpha responses of plasmacytoid dendritic cells to HIV-1. AIDS 25: 715-717.

35. Chaplin B, Eisen G, Idoko J, Onwujekwe D, Idigbe E, et al. (2011) Impact of HIV type 1 subtype on drug resistance mutations in Nigerian patients failing first-line therapy. AIDS Res Hum Retroviruses 27: 71-80.

36. Charpentier C, Talla F, Nguepi E, Si-Mohamed A, Belec L (2011) Virological failure and HIV type 1 drug resistance profiles among patients followed-up in private sector, Douala, Cameroon. AIDS Res Hum Retroviruses 27: 221-230.

37. Chaudhry AA, Botsko M, Weiss L, Egan JE, Mitty J, et al. (2011) Participant characteristics and HIV risk behaviors among individuals entering integrated buprenorphine/naloxone and HIV care. J Acquir Immune Defic Syndr 56 Suppl 1: S14-21.

38. Chege D, Sheth PM, Kain T, Kim CJ, Kovacs C, et al. (2011) Sigmoid Th17 populations, the HIV latent reservoir, and microbial translocation in men on long-term antiretroviral therapy. AIDS 25: 741-749.

39. Chen J, Sun J, Zhang R, Liu L, Zheng Y, et al. (2011) T-SPOT.TB in the diagnosis of active tuberculosis among HIV-infected patients with advanced immunodeficiency. AIDS Res Hum Retroviruses 27: 289-294.

40. Chung MH, Richardson BA, Tapia K, Benki-Nugent S, Kiarie JN, et al. (2011) A randomized controlled trial comparing the effects of counseling and alarm device on HAART adherence and virologic outcomes. PLoS Med 8: e1000422.

41. Clucas C, Harding R, Lampe FC, Anderson J, Date HL, et al. (2011) Doctor-patient concordance during HIV treatment switching decision-making. HIV Med 12: 87-96.

42. Cohen C, Elion R, Ruane P, Shamblaw D, DeJesus E, et al. (2011) Randomized, phase 2 evaluation of two single-tablet regimens elvitegravir/cobicistat/emtricitabine/tenofovir disoproxil fumarate versus efavirenz/emtricitabine/tenofovir disoproxil fumarate for the initial treatment of HIV infection. AIDS 25: F7-12.

43. Cooper DA, Cordery DV, Reiss P, Henry K, Nelson M, et al. (2011) The effects of enfuvirtide therapy on body composition and metabolic parameters over 48 weeks in the TORO body imaging substudy. HIV Med 12: 31-39.

44. Cotter AG, Satchell CS, O'Halloran J A, Feeney ER, Sabin CA, et al. (2011) High-density lipoprotein levels and 10-year cardiovascular risk in HIV-1-infected patients. AIDS 25: 867-869.

45. Cotter EJ, Chew N, Powderly WG, Doran PP (2011) HIV type 1 alters mesenchymal stem cell differentiation potential and cell phenotype ex vivo. AIDS Res Hum Retroviruses 27: 187-199.

46. Crane HM, Grunfeld C, Willig JH, Mugavero MJ, Van Rompaey S, et al. (2011) Impact of NRTIs on lipid levels among a large HIV-infected cohort initiating antiretroviral therapy in clinical care. AIDS 25: 185-195.

47. Crum-Cianflone NF, Eberly LE, Duplessis C, Maguire J, Ganesan A, et al. (2011) Immunogenicity of a monovalent 2009 influenza A (H1N1) vaccine in an immunocompromised population: a prospective study comparing HIV-infected adults with HIV-uninfected adults. Clin Infect Dis 52: 138-146.

48. Crump JA, Ramadhani HO, Morrissey AB, Saganda W, Mwako MS, et al. (2011) Invasive bacterial and fungal infections among hospitalized HIV-infected and HIV-uninfected adults and adolescents in northern Tanzania. Clin Infect Dis 52: 341-348.

49. Curlin ME, Cassis-Ghavami F, Magaret AS, Spies GA, Duerr A, et al. (2011) Serological immunity to adenovirus serotype 5 is not associated with risk of HIV infection: a case-control study. AIDS 25: 153-158.

50. da Silva GK, Guimaraes R, Mattevi VS, Lazzaretti RK, Sprinz E, et al. (2011) The role of mannose-binding lectin gene polymorphisms in susceptibility to HIV-1 infection in Southern Brazilian patients. AIDS 25: 411-418.

51. Dahab M, Kielmann K, Charalambous S, Karstaedt AS, Hamilton R, et al. (2011) Contrasting reasons for discontinuation of antiretroviral therapy in workplace and public-sector HIV programs in South Africa. AIDS Patient Care STDS 25: 53-59.

52. Dao CN, Patel P, Overton ET, Rhame F, Pals SL, et al. (2011) Low vitamin D among HIV-infected adults: prevalence of and risk factors for low vitamin D Levels in a cohort of HIV-infected adults and comparison to prevalence among adults in the US general population. Clin Infect Dis 52: 396-405.

53. D'Aquila RT, Geretti AM, Horton JH, Rouse E, Kheshti A, et al. (2011) Tenofovir (TDF)-selected or abacavir (ABC)-selected low-frequency HIV type 1 subpopulations during failure with persistent viremia as detected by ultradeep pyrosequencing. AIDS Res Hum Retroviruses 27: 201-209.

54. Davies MA, Moultrie H, Eley B, Rabie H, Van Cutsem G, et al. (2011) Virologic failure and second-line antiretroviral therapy in children in South Africa--the IeDEA Southern Africa collaboration. J Acquir Immune Defic Syndr 56: 270-278.

55. de Araujo ES, Dahari H, Cotler SJ, Layden TJ, Neumann AU, et al. (2011) Pharmacodynamics of PEG-IFN-[alpha]-2a and HCV response as a function of IL28B polymorphism in HIV/HCV-coinfected patients. J Acquir Immune Defic Syndr 56: 95-99.

56. Delaney KP, Branson BM, Uniyal A, Phillips S, Candal D, et al. (2011) Evaluation of the performance characteristics of 6 rapid HIV antibody tests. Clin Infect Dis 52: 257-263.

57. Delwart E, Bernardin F, Lee TH, Winkelman V, Liu C, et al. (2011) Absence of reproducibly detectable low-level HIV viremia in highly exposed seronegative men and women. AIDS 25: 619-623.

58. Depatureaux A, Charpentier C, Leoz M, Unal G, Damond F, et al. (2011) Impact of HIV-1 group O genetic diversity on genotypic resistance interpretation by algorithms designed for HIV-1 group M. J Acquir Immune Defic Syndr 56: 139-145.

59. Diaz A, Garcia F, Mozos A, Caballero M, Leon A, et al. (2011) Lymphoid tissue collagen deposition in HIV-infected patients correlates with the imbalance between matrix metalloproteinases and their inhibitors. J Infect Dis 203: 810-813.

60. Djoko CF, Rimoin AW, Vidal N, Tamoufe U, Wolfe ND, et al. (2011) High HIV type 1 group M pol diversity and low rate of antiretroviral resistance mutations among the uniformed services in Kinshasa, Democratic Republic of the Congo. AIDS Res Hum Retroviruses 27: 323-329.

61. Duprez DA, Neuhaus J, Tracy R, Kuller LH, Deeks SG, et al. (2011) N-terminal-proB-type natriuretic peptide predicts cardiovascular disease events in HIV-infected patients. AIDS 25: 651-657.

62. Egan JE, Netherland J, Gass J, Finkelstein R, Weiss L (2011) Patient perspectives on buprenorphine/naloxone treatment in the context of HIV care. J Acquir Immune Defic Syndr 56 Suppl 1: S46-53.

63. Enomoto L, Anderson PL, Li S, Edelstein CL, Weinberg A (2011) Effect of nucleoside and nucleotide analog reverse transcriptase inhibitors on cell-mediated immune functions. AIDS Res Hum Retroviruses 27: 47-55.

64. Fellay J, Frahm N, Shianna KV, Cirulli ET, Casimiro DR, et al. (2011) Host genetic determinants of T cell responses to the MRKAd5 HIV-1 gag/pol/nef vaccine in the step trial. J Infect Dis 203: 773-779.

65. Fiellin DA, Weiss L, Botsko M, Egan JE, Altice FL, et al. (2011) Drug treatment outcomes among HIV-infected opioid-dependent patients receiving buprenorphine/naloxone. J Acquir Immune Defic Syndr 56 Suppl 1: S33-38.

66. Finocchario-Kessler S, Catley D, Berkley-Patton J, Gerkovich M, Williams K, et al. (2011) Baseline predictors of ninety percent or higher antiretroviral therapy adherence in a diverse urban sample: the role of patient autonomy and fatalistic religious beliefs. AIDS Patient Care STDS 25: 103-111.

67. Fitzgerald DW, Janes H, Robertson M, Coombs R, Frank I, et al. (2011) An Ad5-vectored HIV-1 vaccine elicits cell-mediated immunity but does not affect disease progression in HIV-1-infected male subjects: results from a randomized placebo-controlled trial (the Step study). J Infect Dis 203: 765-772.

68. Flor-Parra F, Perez-Pulido AJ, Pachon J, Perez-Romero P (2011) The HIV type 1 protease L10I minor mutation decreases replication capacity and confers resistance to protease inhibitors. AIDS Res Hum Retroviruses 27: 65-70.

69. Fox J, Peters B, Prakash M, Arribas J, Hill A, et al. (2011) Improvement in vitamin D deficiency following antiretroviral regime change: Results from the MONET trial. AIDS Res Hum Retroviruses 27: 29-34.

70. Frank I, Robbiani M (2011) Attachment and fusion inhibitors potently prevent dendritic cell-driven HIV infection. J Acquir Immune Defic Syndr 56: 204-212.

71. Franquelim HG, Chiantia S, Veiga AS, Santos NC, Schwille P, et al. (2011) Anti-HIV-1 antibodies 2F5 and 4E10 interact differently with lipids to bind their epitopes. AIDS 25: 419-428.

72. Fu J, Sha BE, Thomas LL (2011) HIV-1-infected peripheral blood mononuclear cells enhance neutrophil survival and HLA-DR expression via increased production of GM-CSF: implications for HIV-1 infection. J Acquir Immune Defic Syndr 56: 16-25.

73. Gagliani LH, Alkmim Maia WT, Sa-Filho D, Janini LM, Sucupira MC, et al. (2011) The association between primary antiretroviral resistance and HAART virologic failure in a developing set. AIDS Res Hum Retroviruses 27: 251-256.

74. Gallien S, Delaugerre C, Charreau I, Braun J, Boulet T, et al. (2011) Emerging integrase inhibitor resistance mutations in raltegravir-treated HIV-1-infected patients with low-level viremia. AIDS 25: 665-669.

75. Ganesan A, Crum-Cianflone N, Higgins J, Qin J, Rehm C, et al. (2011) High dose atorvastatin decreases cellular markers of immune activation without affecting HIV-1 RNA levels: results of a double-blind randomized placebo controlled clinical trial. J Infect Dis 203: 756-764.

76. Garcia F, Climent N, Assoumou L, Gil C, Gonzalez N, et al. (2011) A therapeutic dendritic cell-based vaccine for HIV-1 infection. J Infect Dis 203: 473-478.

77. Garg S, Hoenig M, Edwards EM, Bliss C, Heeren T, et al. (2011) Incidence and predictors of acute kidney injury in an urban cohort of subjects with HIV and hepatitis C virus coinfection. AIDS Patient Care STDS 25: 135-141.

78. Garrabou G, Lopez S, Moren C, Martinez E, Fontdevila J, et al. (2011) Mitochondrial damage in adipose tissue of untreated HIV-infected patients. AIDS 25: 165-170.

79. Gianotti N, Visco F, Galli L, Barda B, Piatti P, et al. (2011) Detecting impaired glucose tolerance or type 2 diabetes mellitus by means of an oral glucose tolerance test in HIV-infected patients. HIV Med 12: 109-117.

80. Goepfert PA, Elizaga ML, Sato A, Qin L, Cardinali M, et al. (2011) Phase 1 safety and immunogenicity testing of DNA and recombinant modified vaccinia Ankara vaccines expressing HIV-1 virus-like particles. J Infect Dis 203: 610-619.

81. Goodman DD, Zhou Y, Margot NA, McColl DJ, Zhong L, et al. (2011) Low level of the K103N HIV-1 above a threshold is associated with virological failure in treatment-naive individuals undergoing efavirenz-containing therapy. AIDS 25: 325-333.

82. Grabmeier-Pfistershammer K, Steinberger P, Rieger A, Leitner J, Kohrgruber N (2011) Identification of PD-1 as a unique marker for failing immune reconstitution in HIV-1-infected patients on treatment. J Acquir Immune Defic Syndr 56: 118-124.

83. Gras L, van Sighem A, Bezemer D, Smit C, Wit F, et al. (2011) Lower mortality and earlier start of combination antiretroviral therapy in patients tested repeatedly for HIV than in those with a positive first test. AIDS 25: 813-818.

84. Gray R, Ssempiija V, Shelton J, Serwadda D, Nalugoda F, et al. (2011) The contribution of HIV-discordant relationships to new HIV infections in Rakai, Uganda. AIDS 25: 863-865.

85. Guadalupe M, Pollock BH, Westbrook S, Redding S, Bullock D, et al. (2011) Risk factors influencing antibody responses to Kaposi's sarcoma-associated herpesvirus latent and lytic antigens in patients under antiretroviral therapy. J Acquir Immune Defic Syndr 56: 83-90.

86. Haim-Boukobza S, Morand-Joubert L, Flandre P, Valin N, Fourati S, et al. (2011) Higher efficacy of nevirapine than efavirenz to achieve HIV-1 plasma viral load below 1 copy/ml. AIDS 25: 341-344.

87. Hansen AB, Obel N, Nielsen H, Pedersen C, Gerstoft J (2011) Bone mineral density changes in protease inhibitor-sparing vs. nucleoside reverse transcriptase inhibitor-sparing highly active antiretroviral therapy: data from a randomized trial. HIV Med 12: 157-165.

88. Hardy H, Kumar V, Doros G, Farmer E, Drainoni ML, et al. (2011) Randomized controlled trial of a personalized cellular phone reminder system to enhance adherence to antiretroviral therapy. AIDS Patient Care STDS 25: 153-161.

89. Harezlak J, Buchthal S, Taylor M, Schifitto G, Zhong J, et al. (2011) Persistence of HIV-associated cognitive impairment, inflammation, and neuronal injury in era of highly active antiretroviral treatment. AIDS 25: 625-633.

90. Hartigan-O'Connor DJ, Jacobson MA, Tan QX, Sinclair E (2011) Development of cytomegalovirus (CMV) immune recovery uveitis is associated with Th17 cell depletion and poor systemic CMV-specific T cell responses. Clin Infect Dis 52: 409-417.

91. Heath SL, Sabbaj S, Bansal A, Kilby JM, Goepfert PA (2011) CD8 T-cell proliferative capacity is compromised in primary HIV-1 infection. J Acquir Immune Defic Syndr 56: 213-221.

92. Helleringer S, Kohler HP, Kalilani-Phiri L, Mkandawire J, Armbruster B (2011) The reliability of sexual partnership histories: implications for the measurement of partnership concurrency during surveys. AIDS 25: 503-511.

93. Hennig BJ, Velez-Edwards DR, Schim van der Loeff MF, Bisseye C, Edwards TL, et al. (2011) CD4 intragenic SNPs associate with HIV-2 plasma viral load and CD4 count in a community-based study from Guinea-Bissau, West Africa. J Acquir Immune Defic Syndr 56: 1-8.

94. Ho J, Moir S, Wang W, Posada JG, Gu W, et al. (2011) Enhancing effects of adjuvanted 2009 pandemic H1N1 influenza A vaccine on memory B-cell responses in HIV-infected individuals. AIDS 25: 295-302.

95. Horberg M, Hurley L, Towner W, Gambatese R, Klein D, et al. (2011) HIV quality performance measures in a large integrated health care system. AIDS Patient Care STDS 25: 21-28.

96. Howe CJ, Cole SR, Ostrow DG, Mehta SH, Kirk GD (2011) A prospective study of alcohol consumption and HIV acquisition among injection drug users. AIDS 25: 221-228.

97. Hulgan T, Haubrich R, Riddler SA, Tebas P, Ritchie MD, et al. (2011) European mitochondrial DNA haplogroups and metabolic changes during antiretroviral therapy in AIDS Clinical Trials Group Study A5142. AIDS 25: 37-47.

98. Hunt PW, Hatano H, Sinclair E, Lee TH, Busch MP, et al. (2011) HIV-specific CD4+ T cells may contribute to viral persistence in HIV controllers. Clin Infect Dis 52: 681-687.

99. Imamichi H, Degray G, Asmuth DM, Fischl MA, Landay AL, et al. (2011) HIV-1 viruses detected during episodic blips following interleukin-7 administration are similar to the viruses present before and after interleukin-7 therapy. AIDS 25: 159-164.

100. Iser DM, Avihingsanon A, Wisedopas N, Thompson AJ, Boyd A, et al. (2011) Increased intrahepatic apoptosis but reduced immune activation in HIV-HBV co-infected patients with advanced immunosuppression. AIDS 25: 197-205.

101. Israr M, Mitchell D, Alam S, Dinello D, Kishel JJ, et al. (2011) The HIV protease inhibitor lopinavir/ritonavir (Kaletra) alters the growth, differentiation and proliferation of primary gingival epithelium. HIV Med 12: 145-156.

102. Jani IV, Sitoe NE, Chongo PL, Alfai ER, Quevedo JI, et al. (2011) Accurate CD4 T-cell enumeration and antiretroviral drug toxicity monitoring in primary healthcare clinics using point-of-care testing. AIDS 25: 807-812.

103. Jenness SM, Neaigus A, Murrill CS, Wendel T, Forgione L, et al. (2011) Estimated HIV incidence among high-risk heterosexuals in New York City, 2007. J Acquir Immune Defic Syndr 56: 193-197.

104. Joska JA, Westgarth-Taylor J, Hoare J, Thomas KG, Paul R, et al. (2011) Validity of the International HIV Dementia Scale in South Africa. AIDS Patient Care STDS 25: 95-101.

105. Julg B, Moodley ES, Qi Y, Ramduth D, Reddy S, et al. (2011) Possession of HLA class II DRB1*1303 associates with reduced viral loads in chronic HIV-1 clade C and B infection. J Infect Dis 203: 803-809.

106. Kajaste-Rudnitski A, Galli L, Nozza S, Tambussi G, Di Pietro A, et al. (2011) Induction of protective antibody response by MF59-adjuvanted 2009 pandemic A/H1N1v influenza vaccine in HIV-1-infected individuals. AIDS 25: 177-183.

107. Kaplan RC, Sinclair E, Landay AL, Lurain N, Sharrett AR, et al. (2011) T cell activation and senescence predict subclinical carotid artery disease in HIV-infected women. J Infect Dis 203: 452-463.

108. Kaufmann GR, Elzi L, Weber R, Furrer H, Giulieri S, et al. (2011) Interruptions of cART limits CD4 T-cell recovery and increases the risk for opportunistic complications and death. AIDS 25: 441-451.

109. Kok J, Tudo K, Blyth CC, Foo H, Hueston L, et al. (2011) Pandemic (H1N1) 2009 influenza virus seroconversion rates in HIV-infected individuals. J Acquir Immune Defic Syndr 56: 91-94.

110. Koon HB, Fingleton B, Lee JY, Geyer JT, Cesarman E, et al. (2011) Phase II AIDS Malignancy Consortium trial of topical halofuginone in AIDS-related Kaposi sarcoma. J Acquir Immune Defic Syndr 56: 64-68.

111. Korthuis PT, Fiellin DA, Fu R, Lum PJ, Altice FL, et al. (2011) Improving adherence to HIV quality of care indicators in persons with opioid dependence: the role of buprenorphine. J Acquir Immune Defic Syndr 56 Suppl 1: S83-90.

112. Korthuis PT, Tozzi MJ, Nandi V, Fiellin DA, Weiss L, et al. (2011) Improved quality of life for opioid-dependent patients receiving buprenorphine treatment in HIV clinics. J Acquir Immune Defic Syndr 56 Suppl 1: S39-45.

113. Kouyos RD, von Wyl V, Yerly S, Boni J, Rieder P, et al. (2011) Ambiguous nucleotide calls from population-based sequencing of HIV-1 are a marker for viral diversity and the age of infection. Clin Infect Dis 52: 532-539.

114. Kramer MA, Cornelissen M, Paraskevis D, Prins M, Coutinho RA, et al. (2011) HIV transmission patterns among The Netherlands, Suriname, and The Netherlands Antilles: a molecular epidemiological study. AIDS Res Hum Retroviruses 27: 123-130.

115. Kucerova Z, Sokolova OI, Demyanov AV, Kvac M, Sak B, et al. (2011) Microsporidiosis and Cryptosporidiosis in HIV/AIDS Patients in St. Petersburg, Russia: Serological identification of microsporidia and Cryptosporidium parvum in sera samples from HIV/AIDS patients. AIDS Res Hum Retroviruses 27: 13-15.

116. Kwara A, Lartey M, Sagoe KW, Court MH (2011) Paradoxically elevated efavirenz concentrations in HIV/tuberculosis-coinfected patients with CYP2B6 516TT genotype on rifampin-containing antituberculous therapy. AIDS 25: 388-390.

117. Kwong JJ, Cook P, Bradley-Springer L (2011) Improving anal cancer screening in an ambulatory HIV clinic: experience from a quality improvement initiative. AIDS Patient Care STDS 25: 73-78.

118. Labarga P, Soriano V, Caruz A, Poveda E, Di Lello F, et al. (2011) Association between IL28B gene polymorphisms and plasma HCV-RNA levels in HIV/HCV-co-infected patients. AIDS 25: 761-766.

119. Lahtinen A, Kivela P, Hedman L, Kumar A, Kantele A, et al. (2011) Serodiagnosis of primary infections with human parvovirus 4, Finland. Emerg Infect Dis 17: 79-82.

120. Lartey M, Sagoe KW, Yang H, Kenu E, Xexemeku F, et al. (2011) Viral decay rates are similar in HIV-infected patients with and without TB coinfection during treatment with an Efavirenz-based regimen. Clin Infect Dis 52: 547-550.

121. Le Clerc S, Coulonges C, Delaneau O, Van Manen D, Herbeck JT, et al. (2011) Screening low-frequency SNPS from genome-wide association study reveals a new risk allele for progression to AIDS. J Acquir Immune Defic Syndr 56: 279-284.

122. Lehavot K, Huh D, Walters KL, King KM, Andrasik MP, et al. (2011) Buffering effects of general and medication-specific social support on the association between substance use and HIV medication adherence. AIDS Patient Care STDS 25: 181-189.

123. Leider J, Fettig J, Calderon Y (2011) Engaging HIV-positive individuals in specialized care from an urban emergency department. AIDS Patient Care STDS 25: 89-93.

124. Lejeune M, Miro JM, De Lazzari E, Garcia F, Claramonte X, et al. (2011) Restoration of T cell responses to toxoplasma gondii after successful combined antiretroviral therapy in patients with AIDS with previous toxoplasmic encephalitis. Clin Infect Dis 52: 662-670.

125. Lescure FX, Omland LH, Engsig FN, Roed C, Gerstoft J, et al. (2011) Incidence and impact on mortality of severe neurocognitive disorders in persons with and without HIV infection: a Danish nationwide cohort study. Clin Infect Dis 52: 235-243.

126. Lessells RJ, Mutevedzi PC, Cooke GS, Newell ML (2011) Retention in HIV care for individuals not yet eligible for antiretroviral therapy: rural KwaZulu-Natal, South Africa. J Acquir Immune Defic Syndr 56: e79-86.

127. Li JC, Au KY, Fang JW, Yim HC, Chow KH, et al. (2011) HIV-1 trans-activator protein dysregulates IFN-gamma signaling and contributes to the suppression of autophagy induction. AIDS 25: 15-25.

128. Li JF, Lipscomb JT, Wei X, Martinson NA, Morris L, et al. (2011) Detection of low-level K65R variants in nucleoside reverse transcriptase inhibitor-naive chronic and acute HIV-1 subtype C infections. J Infect Dis 203: 798-802.

129. Linas BS, Minkoff H, Cohen MH, Karim R, Cohan D, et al. (2011) Relative time to pregnancy among HIV-infected and uninfected women in the Women's Interagency HIV Study, 2002-2009. AIDS 25: 707-711.

130. Lopez M, Soriano V, Peris-Pertusa A, Rallon N, Restrepo C, et al. (2011) Elite controllers display higher activation on central memory CD8 T cells than HIV patients successfully on HAART. AIDS Res Hum Retroviruses 27: 157-165.

131. Lorente E, Infantes S, Barnea E, Beer I, Garcia R, et al. (2011) TAP-independent human histocompatibility complex-Cw1 antigen processing of an HIV envelope protein conserved peptide. AIDS 25: 265-269.

132. Lubomirov R, Colombo S, di Iulio J, Ledergerber B, Martinez R, et al. (2011) Association of pharmacogenetic markers with premature discontinuation of first-line anti-HIV therapy: an observational cohort study. J Infect Dis 203: 246-257.

133. Macdonell KE, Naar-King S, Murphy DA, Parsons JT, Huszti H (2011) Situational temptation for HIV medication adherence in high-risk youth. AIDS Patient Care STDS 25: 47-52.

134. Macias J, Mira J, Gilabert I, Neukam K, Roldan C, et al. (2011) Combined use of aspartate aminotransferase, platelet count and matrix metalloproteinase 2 measurements to predict liver fibrosis in HIV/hepatitis C virus-coinfected patients. HIV Med 12: 14-21.

135. Madhi SA, Maskew M, Koen A, Kuwanda L, Besselaar TG, et al. (2011) Trivalent inactivated influenza vaccine in African adults infected with human immunodeficient virus: double blind, randomized clinical trial of efficacy, immunogenicity, and safety. Clin Infect Dis 52: 128-137.

136. Magoni M, Scarcella C, Vassallo F, Lonati F, Carosi G, et al. (2011) The evolving burden of HIV infection compared with other chronic diseases in northern Italy. HIV Med 12: 129-137.

137. Malee K, Williams P, Montepiedra G, McCabe M, Nichols S, et al. (2011) Medication adherence in children and adolescents with HIV infection: associations with behavioral impairment. AIDS Patient Care STDS 25: 191-200.

138. Manuel O, Pascual M, Hoschler K, Giulieri S, Alves D, et al. (2011) Humoral response to the influenza A H1N1/09 monovalent AS03-adjuvanted vaccine in immunocompromised patients. Clin Infect Dis 52: 248-256.

139. Martin-Carbonero L, Teixeira T, Poveda E, Plaza Z, Vispo E, et al. (2011) Clinical and virological outcomes in HIV-infected patients with chronic hepatitis B on long-term nucleos(t)ide analogues. AIDS 25: 73-79.

140. Matthews GV, Pham ST, Hellard M, Grebely J, Zhang L, et al. (2011) Patterns and characteristics of hepatitis C transmission clusters among HIV-positive and HIV-negative individuals in the Australian trial in acute hepatitis C. Clin Infect Dis 52: 803-811.

141. McNicholas PM, Mann PA, Wojcik L, Phd PQ, Lee E, et al. (2011) Mapping and characterization of vicriviroc resistance mutations from HIV-1 isolated from treatment-experienced subjects enrolled in a phase II study (VICTOR-E1). J Acquir Immune Defic Syndr 56: 222-229.

142. Meditz AL, MaWhinney S, Allshouse A, Feser W, Markowitz M, et al. (2011) Sex, race, and geographic region influence clinical outcomes following primary HIV-1 infection. J Infect Dis 203: 442-451.

143. Mehta SH, Astemborski J, Kirk GD, Strathdee SA, Nelson KE, et al. (2011) Changes in blood-borne infection risk among injection drug users. J Infect Dis 203: 587-594.

144. Menezes P, Eron JJ, Jr., Leone PA, Adimora AA, Wohl DA, et al. (2011) Recruitment of HIV/AIDS treatment-naive patients to clinical trials in the highly active antiretroviral therapy era: influence of gender, sexual orientation and race. HIV Med 12: 183-191.

145. Merino A, Malhotra R, Morton M, Mulenga J, Allen S, et al. (2011) Impact of a functional KIR2DS4 allele on heterosexual HIV-1 transmission among discordant Zambian couples. J Infect Dis 203: 487-495.

146. Mills EJ, Bakanda C, Birungi J, Mwesigwa R, Chan K, et al. (2011) Mortality by baseline CD4 cell count among HIV patients initiating antiretroviral therapy: evidence from a large cohort in Uganda. AIDS 25: 851-855.

147. Minga AK, Lewden C, Gabillard D, Bomisso GI, Toni TD, et al. (2011) CD4 cell eligibility thresholds: an analysis of the time to antiretroviral treatment in HIV-1 seroconverters. AIDS 25: 819-823.

148. Mitchell C, Hitti J, Paul K, Agnew K, Cohn SE, et al. (2011) Cervicovaginal shedding of HIV type 1 is related to genital tract inflammation independent of changes in vaginal microbiota. AIDS Res Hum Retroviruses 27: 35-39.

149. Mondy KE, Gottdiener J, Overton ET, Henry K, Bush T, et al. (2011) High Prevalence of Echocardiographic Abnormalities among HIV-infected Persons in the Era of Highly Active Antiretroviral Therapy. Clin Infect Dis 52: 378-386.

150. Montes M, Sanchez C, Lewis DE, Graviss EA, Seas C, et al. (2011) Normalization of FoxP3(+) regulatory T cells in response to effective antiretroviral therapy. J Infect Dis 203: 496-499.

151. Mueller SM, Spriewald BM, Bergmann S, Eismann K, Leykauf M, et al. (2011) Influence of major HIV-1 protease inhibitor resistance mutations on CTL recognition. J Acquir Immune Defic Syndr 56: 109-117.

152. Musselwhite LW, Sheikh V, Norton TD, Rupert A, Porter BO, et al. (2011) Markers of endothelial dysfunction, coagulation and tissue fibrosis independently predict venous thromboembolism in HIV. AIDS 25: 787-795.

153. Nattermann J, Vogel M, Nischalke HD, Danta M, Mauss S, et al. (2011) Genetic variation in IL28B and treatment-induced clearance of hepatitis C virus in HIV-positive patients with acute and chronic hepatitis C. J Infect Dis 203: 595-601.

154. Nelson M, Stellbrink HJ, Podzamczer D, Banhegyi D, Gazzard B, et al. (2011) A comparison of neuropsychiatric adverse events during 12 weeks of treatment with etravirine and efavirenz in a treatment-naive, HIV-1-infected population. AIDS 25: 335-340.

155. Nglazi MD, Lawn SD, Kaplan R, Kranzer K, Orrell C, et al. (2011) Changes in programmatic outcomes during 7 years of scale-up at a community-based antiretroviral treatment service in South Africa. J Acquir Immune Defic Syndr 56: e1-8.

156. Nguyen A, Calmy A, Delhumeau C, Mercier IK, Cavassini M, et al. (2011) A randomized crossover study to compare efavirenz and etravirine treatment. AIDS 25: 57-63.

157. Nunes MC, von Gottberg A, de Gouveia L, Cohen C, Moore DP, et al. (2011) The impact of antiretroviral treatment on the burden of invasive pneumococcal disease in South African children: a time series analysis. AIDS 25: 453-462.

158. Omar T, Schwartz S, Hanrahan C, Modisenyane T, Tshabangu N, et al. (2011) Progression and regression of premalignant cervical lesions in HIV-infected women from Soweto: a prospective cohort. AIDS 25: 87-94.

159. Ormsby CE, de la Rosa-Zamboni D, Vazquez-Perez J, Ablanedo-Terrazas Y, Vega-Barrientos R, et al. (2011) Severe 2009 pandemic influenza A (H1N1) infection and increased mortality in patients with late and advanced HIV disease. AIDS 25: 435-439.

160. Ortiz M, Poloni ES, Furrer H, Kovari H, Martinez R, et al. (2011) No longitudinal mitochondrial DNA sequence changes in HIV-infected individuals with and without lipoatrophy. J Infect Dis 203: 620-624.

161. Ott MQ, Barnighausen T, Tanser F, Lurie MN, Newell ML (2011) Age-gaps in sexual partnerships: seeing beyond 'sugar daddies'. AIDS 25: 861-863.

162. Oursler KK, Goulet JL, Crystal S, Justice AC, Crothers K, et al. (2011) Association of age and comorbidity with physical function in HIV-infected and uninfected patients: results from the Veterans Aging Cohort Study. AIDS Patient Care STDS 25: 13-20.

163. Oyaro M, Mbithi J, Oyugi F, Laten A, Anzala O, et al. (2011) Molecular characterization of HIV type 1 among HIV-infected respondents in a cohort being prepared for HIV Phase III vaccine clinical trials, Western Kenya. AIDS Res Hum Retroviruses 27: 257-264.

164. Pacheco AG, Saraceni V, Tuboi SH, Lauria LM, Moulton LH, et al. (2011) Estimating the extent of underreporting of mortality among HIV-infected individuals in Rio de Janeiro, Brazil. AIDS Res Hum Retroviruses 27: 25-28.

165. Padmapriyadarsini C, Ramesh Kumar S, Terrin N, Narendran G, Menon PA, et al. (2011) Dyslipidemia among HIV-infected Patients with tuberculosis taking once-daily nonnucleoside reverse-transcriptase inhibitor-based antiretroviral therapy in India. Clin Infect Dis 52: 540-546.

166. Pando MA, Gomez-Carrillo M, Vignoles M, Rubio AE, dos Ramos Farias MS, et al. (2011) Incidence of HIV type 1 infection, antiretroviral drug resistance, and molecular characterization in newly diagnosed individuals in Argentina: A Global Fund Project. AIDS Res Hum Retroviruses 27: 17-23.

167. Papagno L, Alter G, Assoumou L, Murphy RL, Garcia F, et al. (2011) Comprehensive analysis of virus-specific T-cells provides clues for the failure of therapeutic immunization with ALVAC-HIV vaccine. AIDS 25: 27-36.

168. Papo JK, Bauni EK, Sanders EJ, Brocklehurst P, Jaffe HW (2011) Exploring the condom gap: is supply or demand the limiting factor - condom access and use in an urban and a rural setting in Kilifi district, Kenya. AIDS 25: 247-255.

169. Parekh BS, Hanson DL, Hargrove J, Branson B, Green T, et al. (2011) Determination of mean recency period for estimation of HIV type 1 Incidence with the BED-capture EIA in persons infected with diverse subtypes. AIDS Res Hum Retroviruses 27: 265-273.

170. Pathanapitoon K, Riemens A, Kongyai N, Sirirungsi W, Leechanachai P, et al. (2011) Intraocular and plasma HIV-1 RNA loads and HIV uveitis. AIDS 25: 81-86.

171. Peters PJ, Skarbinski J, Louie JK, Jain S, Roland M, et al. (2011) HIV-infected hospitalized patients with 2009 pandemic influenza A (pH1N1)--United States, spring and summer 2009. Clin Infect Dis 52 Suppl 1: S183-188.

172. Petrovski S, Fellay J, Shianna KV, Carpenetti N, Kumwenda J, et al. (2011) Common human genetic variants and HIV-1 susceptibility: a genome-wide survey in a homogeneous African population. AIDS 25: 513-518.

173. Phanuphak N, Pattanachaiwit S, Pankam T, Pima W, Avihingsanon A, et al. (2011) Active voluntary counseling and testing with integrated CD4 count service can enhance early HIV testing and early CD4 count measurement: experiences from the Thai Red Cross Anonymous Clinic in Bangkok, Thailand. J Acquir Immune Defic Syndr 56: 244-252.

174. Pipkin S, Scheer S, Okeigwe I, Schwarcz S, Harris DH, et al. (2011) The effect of HAART and calendar period on Kaposi's sarcoma and non-Hodgkin lymphoma: results of a match between an AIDS and cancer registry. AIDS 25: 463-471.

175. Polis CB, Gray RH, Bwanika JB, Kigozi G, Kiwanuka N, et al. (2011) Effect of hormonal contraceptive use before HIV seroconversion on viral load setpoint among women in Rakai, Uganda. J Acquir Immune Defic Syndr 56: 125-130.

176. Pop-Eleches C, Thirumurthy H, Habyarimana JP, Zivin JG, Goldstein MP, et al. (2011) Mobile phone technologies improve adherence to antiretroviral treatment in a resource-limited setting: a randomized controlled trial of text message reminders. AIDS 25: 825-834.

177. Price MA, Wallis CL, Lakhi S, Karita E, Kamali A, et al. (2011) Transmitted HIV type 1 drug resistance among individuals with recent HIV infection in East and Southern Africa. AIDS Res Hum Retroviruses 27: 5-12.

178. Prosperi MC, Cozzi-Lepri A, Antinori A, Cassola G, Torti C, et al. (2011) Favourable evolution of virological and immunological profiles in treated and untreated patients in Italy in the period 1998-2008. HIV Med 12: 174-182.

179. Qadir MI, Malik SA (2011) Genetic variation in the HR region of the env gene of HIV: A perspective for resistance to HIV fusion inhibitors. AIDS Res Hum Retroviruses 27: 57-63.

180. Rallon NI, Restrepo C, Naggie S, Lopez M, Del Romero J, et al. (2011) Interleukin-28B gene polymorphisms do not influence the susceptibility to HIV-infection or CD4 cell decline. AIDS 25: 269-271.

181. Raymond AD, Campbell-Sims TC, Khan M, Lang M, Huang MB, et al. (2011) HIV Type 1 Nef is released from infected cells in CD45(+) microvesicles and is present in the plasma of HIV-infected individuals. AIDS Res Hum Retroviruses 27: 167-178.

182. Regidor DL, Detels R, Breen EC, Widney DP, Jacobson LP, et al. (2011) Effect of highly active antiretroviral therapy on biomarkers of B-lymphocyte activation and inflammation. AIDS 25: 303-314.

183. Reynolds SJ, Makumbi F, Nakigozi G, Kagaayi J, Gray RH, et al. (2011) HIV-1 transmission among HIV-1 discordant couples before and after the introduction of antiretroviral therapy. AIDS 25: 473-477.

184. Richert L, Dehail P, Mercie P, Dauchy FA, Bruyand M, et al. (2011) High frequency of poor locomotor performance in HIV-infected patients. AIDS 25: 797-805.

185. Roye ME, Amarakoon, II, Hamilton CL, Eyzaguirre LM, Figueroa P, et al. (2011) Genotypic characterization of HIV type 1 in Jamaica. AIDS Res Hum Retroviruses 27: 91-95.

186. Salgado M, Kirk GD, Cox A, Rutebemberwa A, Higgins Y, et al. (2011) Protective interleukin-28B genotype affects hepatitis C virus clearance, but does not contribute to HIV-1 control in a cohort of African-American elite controllers/suppressors. AIDS 25: 385-387.

187. Sampah ME, Ceccato CM, Blankson JN (2011) HIV type 1-mediated downregulation of HLA-B*57/B*5801 proteins on elite suppressor CD4+ T cells. AIDS Res Hum Retroviruses 27: 183-186.

188. Sanchez V, Masia M, Robledano C, Padilla S, Lumbreras B, et al. (2011) A highly sensitive and specific model for predicting HIV-1 tropism in treatment-experienced patients combining interpretation of V3 loop sequences and clinical parameters. J Acquir Immune Defic Syndr 56: 51-58.

189. Sandler NG, Wand H, Roque A, Law M, Nason MC, et al. (2011) Plasma levels of soluble CD14 independently predict mortality in HIV infection. J Infect Dis 203: 780-790.

190. Satlin MJ, Hoover DR, Glesby MJ (2011) Glycemic control in HIV-infected patients with diabetes mellitus and rates of meeting American Diabetes Association management guidelines. AIDS Patient Care STDS 25: 5-12.

191. Scherrer AU, von Wyl V, Joos B, Klimkait T, Burgisser P, et al. (2011) Predictors for the emergence of the 2 multi-nucleoside/nucleotide resistance mutations 69 insertion and Q151M and their impact on clinical outcome in the Swiss HIV cohort study. J Infect Dis 203: 791-797.

192. Shafer LA, Nsubuga RN, White R, Mayanja BN, Chapman R, et al. (2011) Antiretroviral therapy and sexual behavior in Uganda: a cohort study. AIDS 25: 671-678.

193. Silvestri DM, Modjarrad K, Blevins ML, Halale E, Vermund SH, et al. (2011) A comparison of HIV detection rates using routine opt-out provider-initiated HIV testing and counseling versus a standard of care approach in a rural African setting. J Acquir Immune Defic Syndr 56: e9-32.

194. Singh S, Willig JH, Mugavero MJ, Crane PK, Harrington RD, et al. (2011) Comparative Effectiveness and Toxicity of Statins Among HIV-Infected Patients. Clin Infect Dis 52: 387-395.

195. Sire JM, Vray M, Merzouk M, Plantier JC, Pavie J, et al. (2011) Comparative RNA quantification of HIV-1 group M and non-M with the Roche Cobas AmpliPrep/Cobas TaqMan HIV-1 v2.0 and Abbott Real-Time HIV-1 PCR assays. J Acquir Immune Defic Syndr 56: 239-243.

196. Smith W, Grady C, Krohmal B, Lazovski J, Wendler D (2011) Empirical evaluation of the need for 'on-going consent' in clinical research. AIDS 25: 107-114.

197. Smurzynski M, Wu K, Letendre S, Robertson K, Bosch RJ, et al. (2011) Effects of central nervous system antiretroviral penetration on cognitive functioning in the ALLRT cohort. AIDS 25: 357-365.

198. Soler-Palacin P, Melendo S, Noguera-Julian A, Fortuny C, Navarro ML, et al. (2011) Prospective study of renal function in HIV-infected pediatric patients receiving tenofovir-containing HAART regimens. AIDS 25: 171-176.

199. Soliman EZ, Lundgren JD, Roediger MP, Duprez DA, Temesgen Z, et al. (2011) Boosted protease inhibitors and the electrocardiographic measures of QT and PR durations. AIDS 25: 367-377.

200. Soulie C, Lambert-Niclot S, Wirden M, Simon A, Valantin MA, et al. (2011) Low frequency of HIV-1 tropism evolution in patients successfully treated for at least 2 years. AIDS 25: 537-539.

201. Steenhoff AP, Josephs JS, Rutstein RM, Gebo KA, Siberry GK, et al. (2011) Incidence of and risk factors for community acquired pneumonia in US HIV-infected children, 2000-2005. AIDS 25: 717-720.

202. Sullivan LE, Botsko M, Cunningham CO, O'Connor PG, Hersh D, et al. (2011) The impact of cocaine use on outcomes in HIV-infected patients receiving buprenorphine/naloxone. J Acquir Immune Defic Syndr 56 Suppl 1: S54-61.

203. Swenson LC, Mo T, Dong WW, Zhong X, Woods CK, et al. (2011) Deep sequencing to infer HIV-1 co-receptor usage: application to three clinical trials of maraviroc in treatment-experienced patients. J Infect Dis 203: 237-245.

204. Szep Z, Guaraldi G, Shah SS, Lo Re V, 3rd, Ratcliffe SJ, et al. (2011) Vitamin D deficiency is associated with type 2 diabetes mellitus in HIV infection. AIDS 25: 525-529.

205. Tan DH, Kaul R, Raboud JM, Walmsley SL (2011) No impact of oral tenofovir disoproxil fumarate on herpes simplex virus shedding in HIV-infected adults. AIDS 25: 207-210.

206. Taylor LE, Holubar M, Wu K, Bosch RJ, Wyles DL, et al. (2011) Incident hepatitis C virus infection among US HIV-infected men enrolled in clinical trials. Clin Infect Dis 52: 812-818.

207. Thiebaut R, Matheron S, Taieb A, Brun-Vezinet F, Chene G, et al. (2011) Long-term nonprogressors and elite controllers in the ANRS CO5 HIV-2 cohort. AIDS 25: 865-867.

208. Thirumurthy H, Jafri A, Srinivas G, Arumugam V, Saravanan RM, et al. (2011) Two-year impacts on employment and income among adults receiving antiretroviral therapy in Tamil Nadu, India: a cohort study. AIDS 25: 239-246.

209. Tilghman MW, Guerena DD, Licea A, Perez-Santiago J, Richman DD, et al. (2011) Pooled nucleic acid testing to detect antiretroviral treatment failure in Mexico. J Acquir Immune Defic Syndr 56: e70-74.

210. Topp L, Day CA, Iversen J, Wand H, Maher L (2011) Fifteen years of HIV surveillance among people who inject drugs: the Australian Needle and Syringe Program Survey 1995-2009. AIDS 25: 835-842.

211. Tordato F, Cozzi Lepri A, Cicconi P, De Luca A, Antinori A, et al. (2011) Evaluation of glomerular filtration rate in HIV-1-infected patients before and after combined antiretroviral therapy exposure. HIV Med 12: 4-13.

212. Torian LV, Wiewel EW (2011) Continuity of HIV-related medical care, New York City, 2005-2009: Do patients who initiate care stay in care? AIDS Patient Care STDS 25: 79-88.

213. Tudor D, Bomsel M (2011) The broadly neutralizing HIV-1 IgG 2F5 elicits gp41-specific antibody-dependent cell cytotoxicity in a FcgammaRI-dependent manner. AIDS 25: 751-759.

214. Tungsiripat M, El-Bejjani D, Rizk N, Dogra V, O'Riordan MA, et al. (2011) Carotid intima media thickness, inflammatory markers, and endothelial activation markers in HIV Patients with lipoatrophy increased at 48 weeks regardless of use of rosiglitazone or placebo. AIDS Res Hum Retroviruses 27: 295-302.

215. Van Braeckel E, Bourguignon P, Koutsoukos M, Clement F, Janssens M, et al. (2011) An adjuvanted polyprotein HIV-1 vaccine induces polyfunctional cross-reactive CD4+ T cell responses in seronegative volunteers. Clin Infect Dis 52: 522-531.

216. van Zyl GU, Preiser W, Potschka S, Lundershausen AT, Haubrich R, et al. (2011) Pooling strategies to reduce the cost of HIV-1 RNA load monitoring in a resource-limited setting. Clin Infect Dis 52: 264-270.

217. Venkatesh KK, Madiba P, De Bruyn G, Lurie MN, Coates TJ, et al. (2011) Who gets tested for HIV in a South African urban township? Implications for test and treat and gender-based prevention interventions. J Acquir Immune Defic Syndr 56: 151-165.

218. Vergara-Rodriguez P, Tozzi MJ, Botsko M, Nandi V, Altice F, et al. (2011) Hepatic safety and lack of antiretroviral interactions with buprenorphine/naloxone in HIV-infected opioid-dependent patients. J Acquir Immune Defic Syndr 56 Suppl 1: S62-67.

219. Wang C, Wright TC, Denny L, Kuhn L (2011) Rapid rise in detection of human papillomavirus (HPV) infection soon after incident HIV infection among South African women. J Infect Dis 203: 479-486.

220. Waters L, Fisher M, Winston A, Higgs C, Hadley W, et al. (2011) A phase IV, double-blind, multicentre, randomized, placebo-controlled, pilot study to assess the feasibility of switching individuals receiving efavirenz with continuing central nervous system adverse events to etravirine. AIDS 25: 65-71.

221. Waters LJ, Scourfield AT, Marcano M, Gazzard BG, Bower M, et al. (2011) The evolution of coreceptor tropism in HIV-infected patients interrupting suppressive antiretroviral therapy. Clin Infect Dis 52: 671-673.

222. Ye JR, Xin RL, Bai LS, Lu HY, Yu SQ, et al. (2011) Sequence analysis of the gag-pol gene of human immunodeficiency virus type 1 of intersubtype (B'/C) recombinant strain in Beijing, China. AIDS Res Hum Retroviruses 27: 331-337.

223. Yi T, Cocohoba J, Cohen M, Anastos K, DeHovitz JA, et al. (2011) The impact of the AIDS Drug Assistance Program (ADAP) on use of highly active antiretroviral and antihypertensive therapy among HIV-infected women. J Acquir Immune Defic Syndr 56: 253-262.

224. Zanoni BC, Phungula T, Zanoni HM, France H, Feeney ME (2011) Impact of tuberculosis cotreatment on viral suppression rates among HIV-positive children initiating HAART. AIDS 25: 49-55.

225. Zhang T, He N, Ding Y, Crabtree K, Minhas V, et al. (2011) Prevalence of human herpesvirus 8 and hepatitis C virus in a rural community with a high risk for blood-borne infections in central China. Clin Microbiol Infect 17: 395-401.

226. Zoufaly A, an der Heiden M, Kollan C, Bogner JR, Fatkenheuer G, et al. (2011) Clinical outcome of HIV-infected patients with discordant virological and immunological response to antiretroviral therapy. J Infect Dis 203: 364-371.
